# Supplementary material for: Work Task Association with Lead Urine and Blood Concentrations in Informal Electronic Waste Recyclers in Thailand and Chile
Source: Int J Environ Res Public Health. 2021 Oct 9;18(20):10580. doi: 10.3390/ijerph182010580 (PMC8535566; doi:10.3390/ijerph182010580)
Supplement: Supplementary file 1 [file ijerph-18-10580-s001.zip › ijerph-1404506-supplementary.pdf]

Supplementary Figures

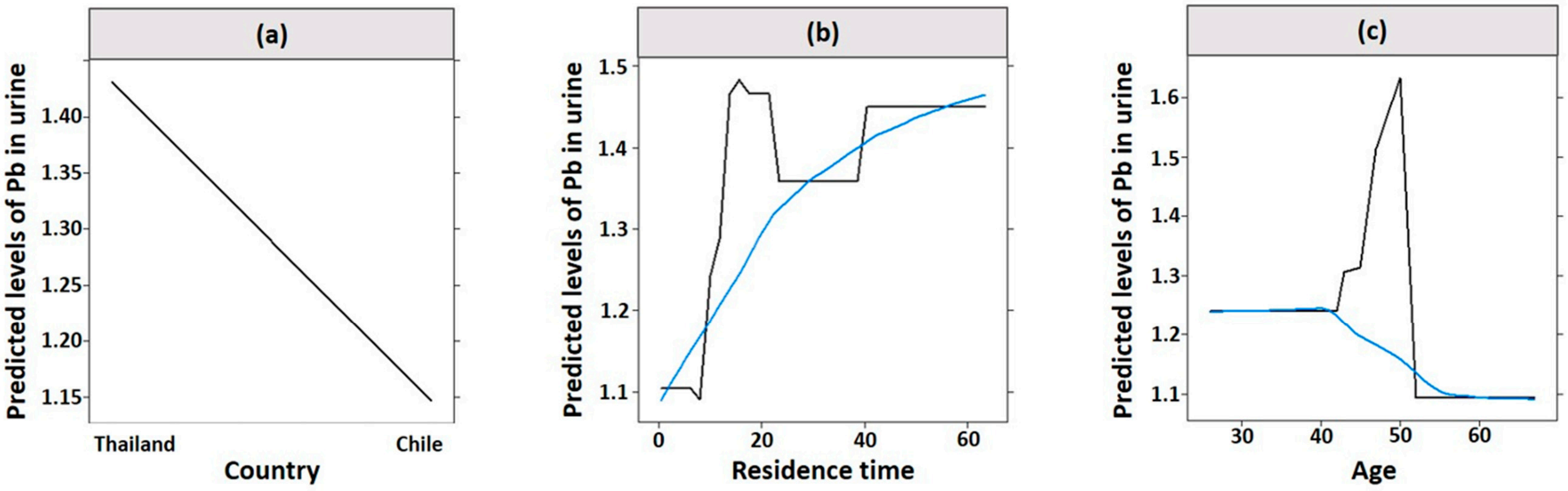

Figure. S1. Partial dependence plots for the most influential demographic factors for log levels of lead (Pb) in urine in order of decreasing relative importance; (a) country; (b) residence time (in years); (c) age. The black line signifies the unsmoothed partial dependence plot, while the blue line signifies the smoothed partial dependence plot.

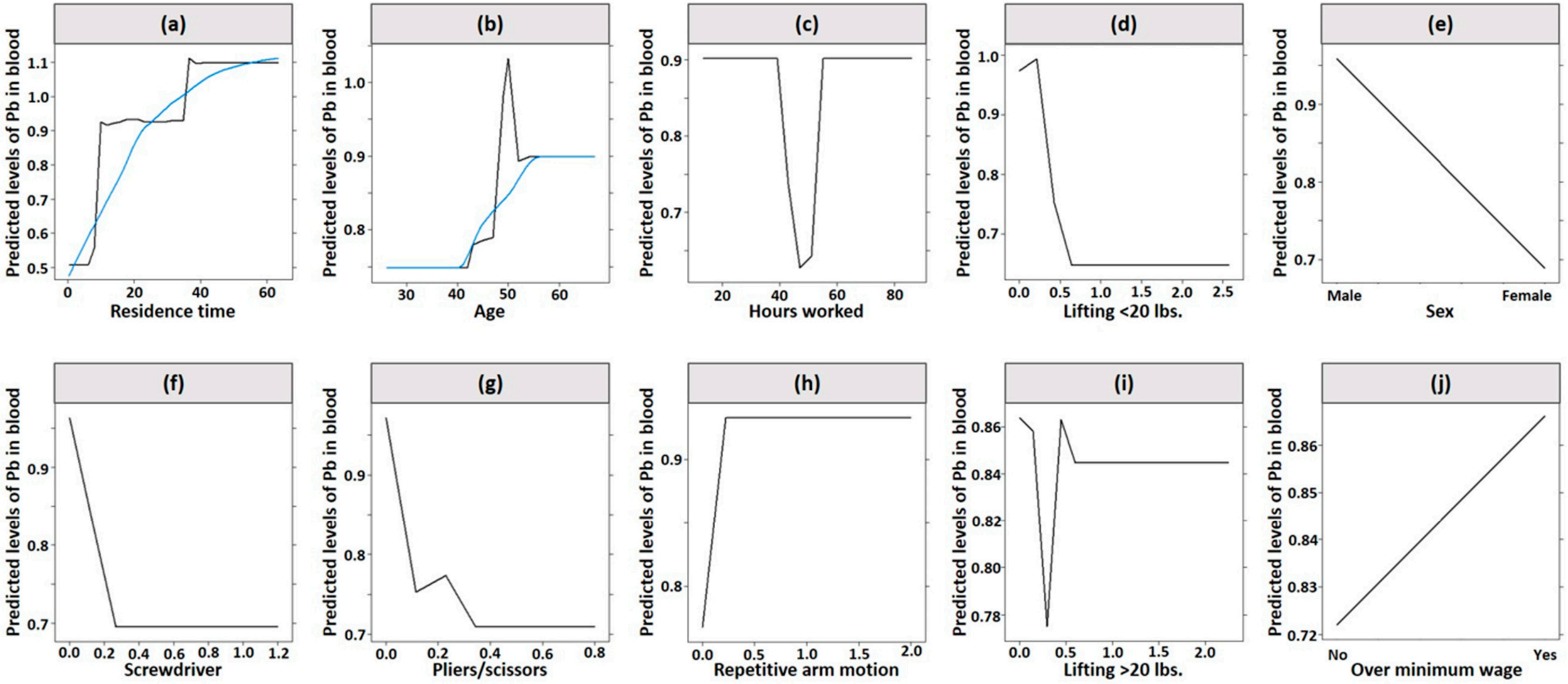

Figure. S2. Partial dependence plots for the most influential demographic and other job factors for log levels of lead (Pb) in blood in order of decreasing relative importance; (a) residence time; (b) age; (c) typical hours of worked per week; (d) lifting objects less than 20 pounds; (e) sex; (f) the use of a screwdriver; (g) the use of pliers/scissors; (h) repetitive arm motion; (i) lifting objects greater than 20 pounds; (j) income greater than minimum wage. The black line signifies the unsmoothed partial dependence plot, while the blue line signifies the smoothed partial dependence plot.
